# Update: factors influencing heart rate variability–a narrative review

**DOI:** 10.3389/fphys.2024.1430458

**Published:** 2024-08-06

**Authors:** Stefan Sammito, Beatrice Thielmann, Irina Böckelmann

**Affiliations:** ^1^ German Air Force Centre of Aerospace Medicine, Cologne, Germany; ^2^ Occupational Medicine, Faculty of Medicine, Otto von Guericke University, Magdeburg, Germany

**Keywords:** autonomic nervous system, heart rate, analysis, sympathetic, parasympathicus

## Abstract

**Objective:**

Heart rate variability (HRV) is an important non-invasive marker for the assessment of an organism’s autonomic physiological regulatory pathways. Lower HRV has been shown to correlate with increased mortality. HRV is influenced by various factors or diseases. The aim of this narrative review is to describe the current state of knowledge on factors influencing HRV and their significance for interpretation.

**Methods:**

The narrative review only included reviews, meta-analyses, and cohort studies which were published until 2021. HRV confounders were grouped into four categories (non-influenceable physiological factors, diseases, influenceable lifestyle factors and external factors).

**Results:**

The review found that HRV was decreased not only in non-influenceable physiological factors (e.g., age, gender, ethnicity) but also in connection with various number of acute and chronic diseases (e.g., psychiatric diseases, myocardial infarction, heart failure), influenceable lifestyle factors (e.g., alcohol abuse, overweight, physical activity), and external factors (e.g., heat, noise, shift work, harmful- and hazardous substances).

**Conclusion:**

In order to improve the quality of HRV studies and to ensure accurate interpretation, it is recommended that confounders be taken into account in future diagnostic measurements or measurements in the workplace (e.g., as part of health promotion measures) in order to counteract data bias.

## 1 Introduction

The measurement and analysis of heart rate variability (HRV), which is based on the variation between consecutive NN intervals, has become an established procedure over the past 2 decades since the publication of the first guideline (Task Force of the European Society of Cardiology and the North American Society of Pacing and Electrophysiology, 1996). Not only have there been advances in recording technology (smaller, more portable, more accurate devices) (Koerber T et al., 2000), but NN intervals can now also be measured by small chest strap and pulse watch systems (Wallén et al., 2012). Technological developments have reduced the costs of recording and analysis and have facilitated outpatient applications. HRV is also becoming increasingly important in clinical medicine, in particular to supplement established diagnostic procedures or to monitor progress. This requires a basic understanding of recording and analysing HRV, for which reference can be made to the relevant guidelines (Task Force of the European Society of Cardiology and the North American Society of Pacing and Electrophysiology, 1996; Sassi et al., 2015; Sammito et al., 2024).

The variability of the successive differences between the NN intervals depends on sympathetic and parasympathetic influences. Mathematical algorithms can be used to calculate various HRV parameters from a time series of successive NN intervals. It is customary to make a distinction between so-called HRV parameters of the time domain and frequency domain and so-called non-linear HRV parameters (Task Force of the European Society of Cardiology and the North American Society of Pacing and Electrophysiology, 1996; Sassi et al., 2015; Sammito et al., 2024).

Increased HRV is generally defined as a state in which the variability of successive cardiac actions is increased or, in the case of reduced variability, HRV is said to be reduced. Depending on the respective HRV parameter, a higher variability (=increased HRV) can be accompanied by a higher value in the respective parameter and *vice versa*, but for some HRV parameters this is the other way round. In addition, some so-called non-linear HRV parameters are based on other mechanisms and are therefore in part less susceptible to external interfering factors.

A decrease in HRV has been shown to correlate with increased mortality, for example, after myocardial infarction (Buccelletti et al., 2009; Huikuri and Stein, 2013; Song et al., 2014), strokes (Yperzeele et al., 2015), bypass operations (Lakusic et al., 2013), heart failure (Sandercock and Brodie, 2006), or chronic obstructive pulmonary disease (Handa et al., 2012). An association could also be shown for manifestation of hypertension 3 years later if the HRV was decreased (Liao et al., 1996; Singh et al., 1998; Schroeder et al., 2003).

The HRV analysis can be performed based on both a short-term (5 min, sometimes shorter) and a long-term measurement (usually 24 h) (Sammito et al., 2024). Although the analysis windows are different, the reduction of HRV in underlying diseases is evident in both the short-term and long-term measurements. While intra-individual comparisons are usually uncomplicated, such confounders play a role when inter-individual comparisons are to be made between individuals or groups. In this case, it is important to know possible influencing factors and their effect on HRV.

## 2 Methods

The group of authors published a first review on factors influencing heart rate variability in 2016 (Sammito and Böckelmann, 2016). Since then, a number of new findings have been added, making an update of this work urgently necessary. Based on an updated narrative review the authors of this article have included known literature to this topic supplemented by information from national and international guidelines (Task Force of the European Society of Cardiology and the North American Society of Pacing and Electrophysiology, 1996; Sassi et al., 2015; Sammito et al., 2024), and presented the HRV confounders grouped in four categories (uncontrollable physiological factors, diseases, controllable lifestyle factors and external factors). Each search included the search terms “HRV” or “heart rate variability” and each confounder or superordinate term, e.g., heart disease. References primarily include meta-analyses and systematic reviews on the topic, supplemented by cohort studies. Published articles were considered if they were written in English or German and were published up to the end of 2021.

## 3 Results

In addition to non-influenceable physiological parameters, a number of factors come from the lifestyle habits of the test persons, from the consequences of these habits and from external circumstances. A number of diseases are associated with a decrease in HRV, while the influence on the vegetative nervous system can be regarded more as a result of diseases and only rarely as a potential cause of these decrease.

### 3.1 Physiological factors

Non-influenceable physiological factors include age, sex, pregnancy and circadian rhythm.

A person’s HRV first increases sharply until they reach the age of 1 year and continues to increase considerably until they reach the age of 15 years, while the resting heart rate decreases (Eyre et al., 2014). The HRV is highest in young adulthood and decreases nonlinearly with age (Umetani et al., 1998; Fagard et al., 1999; Kuo et al., 1999; Fukusaki et al., 2000; Fagard, 2001; Ferrari, 2002; Felber Dietrich et al., 2006; Britton et al., 2007; Zhang, 2007; Barantke et al., 2008; Greiser et al., 2009; Stein et al., 2009; Voss et al., 2009; Shiogai et al., 2010; Haerting et al., 2012; Voss et al., 2012; Abhishekh et al., 2013; Alvares et al., 2016).

Furthermore, there is a difference between women and men, with most studies showing higher parasympathetic activity shown in most studies in women compared to men (Tsuji et al., 1996; Jensen-Urstad et al., 1997; Agelink et al., 2001; Snieder et al., 2007; Barantke et al., 2008; Sookan and McKune, 2012; Abhishekh et al., 2013; Koenig and Thayer, 2016), which however showed a smaller difference after the age of 50 years (Fagard et al., 1999; Kuo et al., 1999; Fagard, 2001). This circumstance seems to be related to the postmenopausal change in the hormonal situation in women (Huikuri et al., 1996; Fagard, 2001). Some of the studies showed a higher baseline sympathetic activity in women (Ramaekers et al., 1998; Umetani et al., 1998; Felber Dietrich et al., 2006; Huang et al., 2012). In summary, it can be assumed that there is a difference in HRV between men and women up to the age of 50 years and therefore gender must be considering as a confounding factor when interpreting HRV.

HRV, like a number of other physiological parameters, is subject not only to age and gender, but also to a circadian rhythm (Sammito et al., 2016). HRV increases during the night and decreases considerably during the morning hours. This must be taken into account, particularly for short-term measurements of a few minutes to a few hours, since intra- and interpersonal comparisons of short-term measurements can only be meaningful if the same time of day is taken into account.

While a genetic disposition of the HRV has been discussed in twin studies (Riese et al., 2007), Riese et al. (2014) found no association between eight key genes for the presence of acetylcholine receptors as part of the autonomic nervous system and the HRV level in an analysis of several cohort studies involving a total of 6,470 test persons. In contrast, ethnicicity seems to have an influence on HRV. In a meta-analysis based on a systematic reference survey involving 17 studies and a total of 11,162 test persons, Hill et al. (2015) established a significantly higher short-term resting HRV in African-American test persons than in American subjects of European origin.

### 3.2 Diseases and health impairments

The effects of various diseases on HRV have been examined in many studies. HRV is consistently lower in patients with these diseases than in healthy test persons. What´s certain is that a low HRV can be found in patients with cardiovascular diseases like cardiac insufficiency (Scalvini et al., 1998; Biswas et al., 2000; Guzzetti et al., 2001; Davies et al., 2002; Lasisi et al., 2012), hypertension (Silvetti et al., 2001; Carthy, 2014), coronary heart disease (CHD) with and without angina pectoris and after myocardial infarction (Huikuri and Mäkikallio, 2001; Huikuri and Stein, 2013).

Also, patients with metabolic disorders also show reduced HRV. Metabolic syndrome often leads to a reduction of the HRV (Liao et al., 1998; Hemingway et al., 2005; Stein et al., 2007; Min et al., 2008; Gehi et al., 2009; Koskinen et al., 2009; Assoumou et al., 2010; Chang et al., 2012), especially in women (Stuckey et al., 2014). HRV is also reduced in manifest diabetes mellitus (Tsuji et al., 1996; Singh et al., 2000; Karayannis et al., 2012; Kuehl and Stevens, 2012; Benichou et al., 2018), although a correlation between disease duration and HRV reduction is only found in very poorly controlled diabetes mellitus (Stein et al., 2007). This is mainly due to peripheral neuropathy caused by microcirculatory disturbances (Barrett et al., 2017).

Reduced HRV is also evident in numerous psychiatric disorders. Patients with anorexia nervosa (Chalmers et al., 2014), anxiety disorders (Aasman et al., 1987; Friedman, 2007; Chalmers et al., 2014; Alvares et al., 2016; Paniccia et al., 2017), bipolar disorder (Alvares et al., 2016; Bassett, 2016; Faurholt-Jepsen et al., 2017; Carr et al., 2018), borderline personality disorder (Koenig et al., 2016b), bulimia nervosa (Peschel et al., 2016) (major) depression (Birkhofer et al., 2005; Kemp et al., 2010; Kapfhammer, 2011; Stapelberg et al., 2012; Alvares et al., 2016; Bassett, 2016; Brown et al., 2018), epilepsy (Lotufo et al., 2012), panic attacks (Aasman et al., 1987; Friedman and Thayer, 1998), posttraumatic stress disorder (Sammito et al., 2015) and schizophrenia (Clamor et al., 2016) have typically shown reduced HRV. In the case of substance addiction (Alvares et al., 2016), the HRV is also usually reduced.

There is also evidence for several other diseases that the HRV is reduced in patients with this diagnosis, such as chronic obstructive pulmonary disease (COPD) (Roque et al., 2014; Mohammed et al., 2015), chronic kidney failure (Zhang and Wang, 2014), in the early stages of Duchenne muscular dystrophy and in manifest disease (da Silva et al., 2018), regular headaches (Barloese, 2016; Koenig et al., 2016c), chronic pain (Koenig et al., 2016a; Tracy et al., 2016), and long-/post-covid (Suh et al., 2023). There is also scientific evidence that HRV is reduced in burnout symptoms (Thielmann et al., 2021; Wekenborg et al., 2022).

However, it is important to remember that for some diseases there is no scientific evidence for a reduced HRV. So, the influence of breast cancer on HRV is unclear (Arab et al., 2016) and based on a systematic literature search, HRV does not currently appear to be changed in the presence of rheumatoid arthritis (Adlan et al., 2014). A reduction in HRV in the presence of sleep disorders is currently not supported, too, by the scientific literature (Dodds et al., 2017). Something similar can be found in untreated obstructive sleep apnea syndrome.

### 3.3 Influenceable lifestyle factors

In the scientific literature, there is a basically consistent picture of the modifiable lifestyle factors: positively associated lifestyle factors, which go hand in hand with a healthy lifestyle, increase the HRV, while negatively associated lifestyle factors reduce it. Thus, the HRV is usually reduced in situations of acute alcohol consumption (Ralevski et al., 2019). A low, constant alcohol consumption with an alcohol content of one standard drink for women or two standard drinks for men usually leads to a short-term but no long-term change in HRV or an increased HRV, while chronic alcohol abuse leads to a reduction of HRV (Karpyak et al., 2014; Ralevski et al., 2019). Increased body mass index (BMI) and increased mass of body fat often cause a fall in the HRV (Fraley et al., 2005). In regard to physical activity, initially, there is a decrease in the HRV due to increased activity of the sympathetic system (Bernardi and Piepoli, 2001), but regular physical activity leads to an increase in the parasympathetic activity which in turn causes a rise in HRV (Bernardi and Piepoli, 2001; Braith and Edwards, 2003; Rennie et al., 2003; Felber Dietrich et al., 2006; Hottenrott et al., 2006; Grässler et al., 2021). Endurance training normally increases the HRV (Aubert et al., 2003; Sandercock et al., 2005; Hottenrott et al., 2006; Routledge et al., 2010; Bellenger et al., 2016; Grässler et al., 2021). Endurance, coordinative, and multimodal training increase HRV in older adults but not resistance training (Grässler et al., 2021). These effects can be also seen in patients with myocardial infarction and patients with heart failure (Routledge et al., 2010) or diabetes mellitus II (Bhati et al., 2018). Similar effects could be observed in individuals who perform high-intensity interval training (HIIT) which generally increases HRV and has been shown to be particularly effective in healthy subjects (Grässler et al., 2021) and patients with metabolic syndrome (Abreu et al., 2019). In contrast, high-intensity training and competition series, on the other hand, can lead to reduced HRV (Aubert et al., 2003; Hottenrott et al., 2006). During strength training, there is usually no change in HRV in healthy individuals, while strength training is usually associated with an increase in HRV in subjects with chronic illnesses (Bhati et al., 2019).

Other lifestyle habits such as smoking can lead to a dose-dependent decrease in HRV (Felber Dietrich et al., 2007; Dinas et al., 2013). Even in non-smokers, passive smoking, e.g., at home or at work leads to a reduction in the HRV (Felber Dietrich et al., 2007; Wilson et al., 2010; Dinas et al., 2013). Stress (e.g., mental, work-related) generally leads to decreased parasympathetic activity and thus to a reduction in the HRV (Dishman et al., 2000; Lehrer, 2003a; Chandola et al., 2008; Chandola et al., 2010; Looser et al., 2010; Clays et al., 2011; Järvelin-Pasanen et al., 2018).

HRV have been used for biofeedback in cases of stress recovery and recently also in the treatment of posttraumatic stress disorder, e.g., for an objective view on the effects of stress relaxation (Lehrer et al., 2003; Lehrer et al., 2003; Del Pozo et al., 2004; Lehrer et al., 2006; Peira et al., 2013). However, until now, only short-term effects of such interventions have been observed. It has not yet been possible to demonstrate a long-term effect (Peira et al., 2013). Nevertheless, biofeedback should be considered as a possible confounder.

### 3.4 External factors

The effects of respiration on HRV are reflected in the form of respiratory sinus arrhythmia (RSA) and is seen in the HF band. On the whole, the HRV parameter, Root Mean Square of Successive Differences (RMSSD), does not seem to be affected by respiration (Hill and Siebenbrock, 2009). For the rest of the parameters, the present state of knowledge is not conclusive (Jennings and Mack, 1984; Kanters et al., 1997; Schaffer et al., 2014).

In addition to climatic conditions and work-related parameters, several harmful substances and medications also have a direct or indirect influence on HRV. Climatic factors lead to changes in HRV due to the physiological response of the vegetative nervous system. Heat increases the activity of the sympathetic nervous system activity, which reduces the HRV (Ren et al., 2011; Wu et al., 2013). Long-term exposure to cold (e.g., at work or during the winter months) was found to have no effect on HRV (Harinath et al., 2005; Bortkiewicz et al., 2006; Ren et al., 2011) due to adaptation effects, e.g., after 60 days. Hypobaric hypoxia usually leads to short-term sympathetic activation (Bhaumik et al., 2013) and long-term to a reduction in HRV (Dhar et al., 2014). Noise exposure also decrease HRV by increasing sympathetic nervous system activity (Lee et al., 2010; Kraus et al., 2013; Schnell et al., 2013; Veternik et al., 2018).

Shift work with a night shift usually results in an activation of the sympathetic nervous system (SNS) and a reduction of the parasympathetic nervous system (PNS) and thus a reduction in HRV, whereby there is a correlation between the duration of shift work in years and the reduction of HRV (Ha et al., 2001; Chung et al., 2009; Lindholm et al., 2012; Wehrens et al., 2012; Järvelin-Pasanen et al., 2013; Amirian et al., 2014; Jensen et al., 2016).

Some harmful substances (including acute diesel and biodiesel inhalation (Brito et al., 2010), chronic exposure to lead (Murata et al., 1995; Böckelmann et al., 2002), acute exposure to cadmium (Feng et al., 2015), carbon disulfide (Bortkiewicz et al., 1997; Jhun et al., 2003), however, not in the case of long-term low-dose exposure (Reinhardt et al., 1997); long-term mercury exposure (Grandjean et al., 2004), especially as a fetal mercury exposure (Grandjean et al., 2004) and neurotoxic styrene (Murata et al., 1991a; Murata et al., 1991b), and some medications (e.g., beta-blockers, ACE inhibitors, antiarrhythmics and psychotropic drugs) have been found to have a direct or indirect influence on HRV.

**FIGURE 1:**
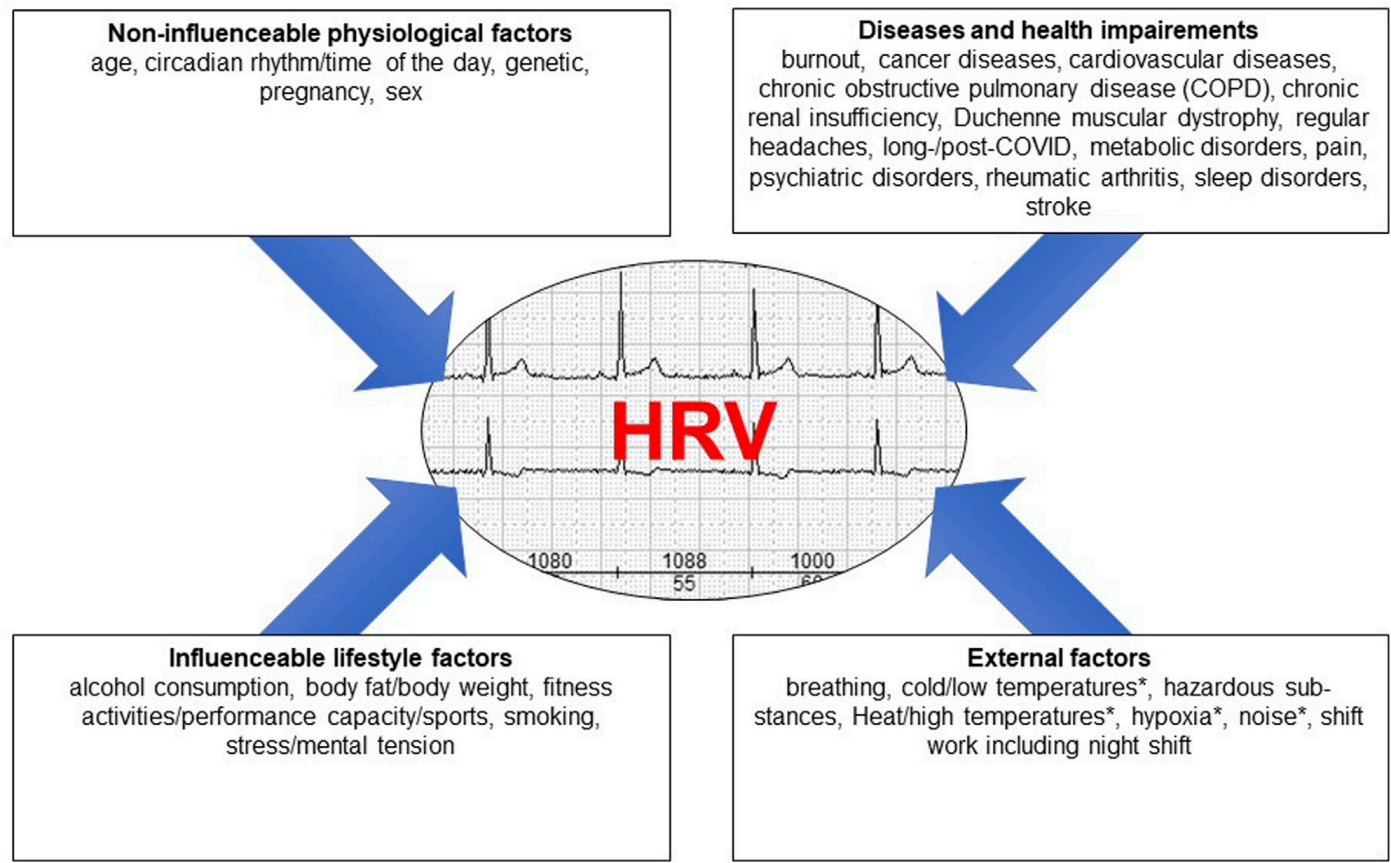
The different factors influencing HRV grouped into four main areas, * = HRV decrease as a result of a physiological reaction to a physical stimulus.

Figure 1 provides a summary of the results referring to the factors and covers the four main categories, i.e., non-influenceable physiological factors, diseases, influenceable lifestyle factors, and external factors.

## 4 Discussion

A decrease in HRV has been observed not only in association with non-influenceable physiological factors such as age, gender, and ethnicity, but also in association with a variety of acute and chronic diseases. Numerous lifestyle factors have both a positive and a negative effects on HRV. There are also physical influences that affect HRV. These should be recognized when analyzing HRV in intra- and interpersonal comparisons. Although not all of the factors on the list have yet been fully researched, awareness of the many factors is of crucial importance in the measurement of HRV (both under laboratory conditions and during medical practice), its analysis and its assessment.
